# Galactomannan, a Surrogate Marker for Outcome in Invasive Aspergillosis: Finally Coming of Age

**DOI:** 10.3389/fmicb.2018.00661

**Published:** 2018-04-04

**Authors:** Toine Mercier, Ellen Guldentops, Katrien Lagrou, Johan Maertens

**Affiliations:** ^1^Laboratory of Clinical Bacteriology and Mycology, Department of Microbiology and Immunology, KU Leuven, Leuven, Belgium; ^2^Department of Hematology, University Hospitals Leuven, Leuven, Belgium; ^3^Department of Laboratory Medicine and National Reference Center for Mycosis, University Hospitals Leuven, Leuven, Belgium

**Keywords:** galactomannan, kinetics, invasive aspergillosis, prognosis, outcome

## Abstract

Detection of galactomannan has become widely available for diagnosing invasive aspergillosis. The test characteristics, using the Platelia™ enzyme-immunoassay, have been well described. This assay could potentially also be useful for the early evaluation of the efficacy of antifungal therapy and for predicting the outcome in terms of response and survival. In this systematic review, we assessed the available evidence for the use of serum galactomannan at baseline as a prognostic marker, and the predictive value of serum galactomannan kinetics after initiation of antifungal therapy. Overall, serum galactomannan at baseline and galactomannan kinetics appear to be good predictors of therapy response and survival. However, breakpoints for predicting therapy failure and validation in different patient populations are still lacking.

## Introduction

Invasive aspergillosis (IA) is a potentially life-threatening disease, occurring mostly in severely immunocompromised patients such as those with acute myeloid leukemia, those with prolonged neutropenia due to myelotoxic therapy, or following allogeneic hematopoietic cell transplantation or solid organ transplantation, and is estimated to affect around 200,000 patients per year (Brown et al., 2012). Timely initiation of therapy is important for improved survival, but diagnosis remains notoriously difficult, especially when relying on conventional culture or microscopy (Lamoth and Calandra, 2017). Because of this, new biomarkers for early diagnosis of IA have been introduced over the last 2 decades. We have summarized the advantages and disadvantages of these tests in Table 1. The diagnostic performance of these biomarkers can be further improved by using them as a combination of tests (Aguado et al., 2015; Neofytos et al., 2015).

**Table 1:** A summarized overview of diagnostic tests in invasive aspergillosis.

	**GM**	**PCR**	**LFD**	**β-D-Glucan**
Early detection possible	+	+	+	+
Broad range of pathogens detected	−	±	−	+
Identification to species level	−	+	−	−
Good performance	+	+	+	+
Quantitative results	+	±	−	+
Rapidly available	+	+	++	+
Low cost	± (in house or referral)	−	?	−

*GM, galactomannan; PCR, polymerase chain reaction; LFD, lateral flow device*.

**Figure 1:**
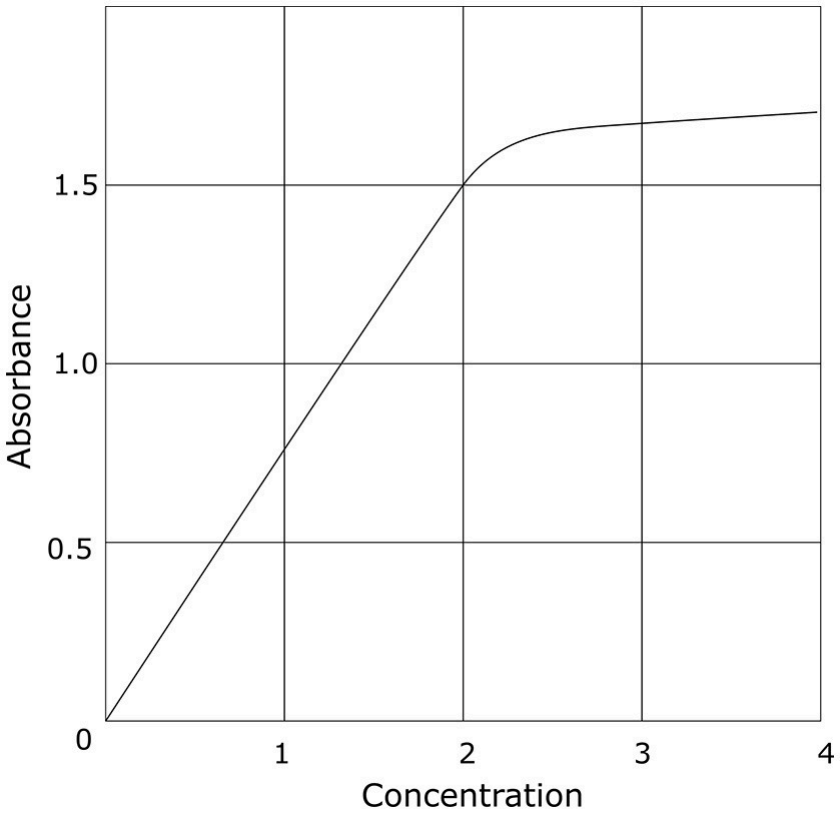
Idealized example of the relation between concentration and optical density in photometry. This is a generalization which holds true for all photometers. The specific numbers will be different depending on the device used.

Galactomannan (GM) belongs to a group of polysaccharides which consist of a mannose backbone and a variable number of galactofuran side chains. GM makes up a major part of the cell wall of *Aspergillus* spp. (Latgé et al., 1994). These galactofuranose-containing polysaccharides vary in size from 35 to 200 kDa and are secreted *in vivo* by the fungus during invasive growth. In recent years, the detection of galactofuranose-containing antigens, including GM, has been used for diagnosing invasive aspergillosis (IA). To date, the most commonly used method to determine GM in serum and broncho-alveolar lavage (BAL) fluid is a double sandwich enzyme-linked immune assay (Platelia™ Aspergillus antigen, Bio-Rad, Marnes-la-Cocquette, France). This assay is based on the rat-derived EB-A2 monoclonal IgM antibody, which acts as capture and detector antibody, and which selectively binds to four or more β(1 → 5) galactofuranosyl residues of GM (Mennink-Kersten et al., 2004). This assay is approved by the US Food and Drug Administration, commercially available, and has been incorporated as a microbiological criterion in the European Organization for Research and Treatment of Cancer-Mycosis Study Group consensus definitions of invasive fungal disease (Pauw et al., 2008). Although this assay has been approved for use in serum and BAL fluid only, successful determination of GM in other matrices such as cerebrospinal fluid (Chong et al., 2016), urine (Reischies et al., 2016), plasma (White et al., 2013), and fluid from abscesses (Verweij et al., 2000) has been reported as well. Results are reported as an optical density index (ODI), where the absorbance value of a clinical sample is compared to the mean of two reference samples (the cut-off controls) provided by the manufacturer. However, absorbance levels are only reliable within a given interval, depending on the type of photometer that is used. This represents a major limitation of the assay. At higher optical densities, the relation between the concentration of GM and the absorbance value becomes non-linear (Figure 1), resulting in the underestimation of concentrations above the linear range. Since the optical density of the reference standards can vary between assay runs, the cutoff at which the assay turns non-linear can also be variable. According to the manufacturer's instruction, the mean optical density of the cut-off controls has to be ≥0.300 and ≤ 0.800. For example, a good quality photometer with a linear range up to an absorbance of 2.5 will therefore be able to accurately report an ODI between 8.33 (for a mean cut-off control of 0.300) and 3.13 (for a mean cut-off control of 0.800). In a lower quality photometer with a linear range up to an absorbance of 1.0, this limit of reliable quantification can be as low as 1.25 (for a mean cut-off control of 0.800). As such, small variations of high ODI's should be interpreted with caution. For an accurate determination of higher values of GM (outside the linear range), the ELISA should be repeated in serially diluted samples, or other, more accurate methods such as mass-spectrometry should be used. Currently, the manufacturer recommends a cut-off of 0.5 in both serum and BAL. However, due to the large number of false positives in BAL at this cutoff, a higher cutoff of 1.0 is proposed in the upcoming revision of the EORTC-MSG criteria.

The test characteristics and limitations of GM detection for diagnosing IA have been well studied and have been the subject of several meta-analyses (Pfeiffer et al., 2006; Zou et al., 2012; Leeflang et al., 2015). Besides providing information on diagnosis, serum GM (sGM) has also been explored for predicting the outcome after initiation of treatment, in particular because the test is easy to perform, widely available, largely *Aspergillus* specific, standardized, and objective. However, the sGM concentration *in vivo* is determined not only by the rate of production and secretion by the growing fungus, but also by the rate of uptake in the bloodstream, as well as the rate of elimination from the circulation.

Due to the relative large size of GM, the antigen cannot freely diffuse from the alveoli through the endothelial lining of the pulmonary capillaries; angio-invasion is required to reach the circulation. This was confirmed in an *in vitro* model of human alveoli, in which GM only appeared in the bloodstream after invasive growth of *Aspergillus* through the alveolar-capillary membrane (Hope et al., 2007). Obviously, as clearly demonstrated in histopathological studies and studies using quantitative polymerase chain reaction (PCR), the degree of angio-invasion (and hence fungal burden) varies with the nature of the underlying condition, with massive invasion and a high fungal burden in neutropenic models and predominantly inflammation with little invasion and a low fungal burden in steroid-induced models (Sheppard et al., 2006). Production of GM is further influenced by therapy; this explains the decreased sensitivity of sGM detection in patients receiving mold-active therapy (Leeflang et al., 2015). This finding was confirmed in animal models, where a concentration-dependent effect on sGM detection was shown for triazoles, polyenes, and investigational drugs such as the orotomides (Petraitiene et al., 2001; Petraitis et al., 2016; Kimura et al., 2017; Negri et al., 2017). One model showed a paradoxical increase in sGM after treatment with caspofungin (Petraitiene et al., 2002), potentially due to interference with fungal cell wall synthesis. However, other models using echinocandins could not replicate this phenomenon (Miceli and Anaissie, 2007). It is more likely that the “paradoxical effect” was caused by ineffective therapy resulting in increased fungal load, rather than an increased release from the cell wall, as echinocandins have been shown to have limited activity against *Aspergillus* spp. in humans (Viscoli et al., 2009). In most comparative animal models, no difference in sGM kinetics was seen between different antifungal drugs when compared at the same level of efficacy.

Elimination of sGM occurs via different routes *in vivo*. Using radioactively labeled *A. fumigatus* GM, a rat and rabbit model of IA showed liver concentration of about one third of the initially injected dose via uptake in Kupffer cells (Bennett et al., 1987). The macrophage mannose receptor plays a central role in this process as hepatic uptake was decreased upon administration of inhibitors of this receptor (Bennett et al., 1987). Another third was excreted renally within 24 h, which is in line with the appearance of GM in urine of patients with IA (Reischies et al., 2016). Renal clearance also depends on the renal function (and the size of the molecule), as is further evidenced by a case report of IA in a patient on hemodialysis who had increasing sGM levels, despite adequate treatment and clinical improvement (Saleeby et al., 2005). Finally, neutrophils are also believed to be involved in the uptake and elimination of circulating GM. This would explain the significantly higher sensitivity of sGM detection in neutropenic patients compared to non-neutropenic ones (Pfeiffer et al., 2006). In addition, a rabbit model confirmed that lower levels of sGM appear in non-neutropenic rabbits, compared to neutropenic rabbits, while no difference in GM could be found in BAL fluid (Petraitiene et al., 2015). Therefore, the interaction between production and secretion during invasive growth, size of the fungal burden, anti-mold therapy, renal and hepatic function, and neutropenic state, results in a complex kinetic profile for sGM.

To determine the current state of the art of the role of GM and its kinetics in the outcome of IA, we searched the MEDLINE database through Pubmed using the following structured query: (“galactomannan”[Supplementary Concept] OR “galactomannan”[All Fields]) AND (“prognosis”[MeSH Terms] OR “prognosis”[All Fields] OR response [All Fields] OR “therapy”[Subheading] OR “therapy”[All Fields] OR “treatment”[All Fields] OR “therapeutics”[MeSH Terms] OR “therapeutics”[All Fields] OR “outcome”[All Fields]). From a total of 911 articles, 56 articles were selected based on title and abstract.

## Kinetics in humans

**Figure 2:**
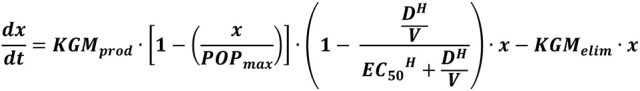
A pharmacokinetic model for serum galactomannan in invasive aspergillosis, as proposed by Huurneman et al. (2016). The first part of the equation estimates production of galactomannan, taking into account the effect of antifungal therapy, whereas the second part estimates the elimination from the bloodstream. x, serum galactomannan; KGM_prod_, maximal rate of galactomannan production; POP_max_, maximal achievable galactomannan; D, drug concentration in the central compartment; V, volume of the central compartment; H, relationship between drug concentration and reduction in galactomannan production; EC_50_, drug concentration at which half-maximal reduction in galactomannan production is reached; KGM_elim_, maximal rate of elimination of galactomannan.

We failed to identify any data on the kinetics of sGM after its administration to healthy volunteers, which would allow us a detailed exploration of its kinetics and metabolism. However, different sources of false positivity (such as GM-containing electrolyte solutions or beta-lactam antibiotics) allow some insight into its kinetics in the human body. One study looked at sGM after infusion of beta-lactam antibiotics in patients who were previously GM seronegative and who were deemed not to have IA based on clinico- radiological signs and symptoms (Aubry et al., 2006). After infusion, a sudden increase in sGM was seen. Based on the declining sGM levels thereafter, the authors estimated a serum half-life of 2.4 days for eliminating sGM. However, influencing parameters such as creatinine clearance and neutrophil count were not reported. Huurneman et al proposed a pharmacokinetic model for the evolution of sGM during antifungal therapy (Figure 2), based on a small number of pediatric patients with IA receiving voriconazole with therapeutic drug monitoring (Huurneman et al., 2016). This model showed a good fit with the actual values, but was limited by the very small number of actual sGM measurements, inclusion of possible cases of IA, and by not taking into account the three different metabolic routes (kidney, liver, and neutrophils).

## Impact of GM at baseline on outcome

We identified 16 studies that looked at GM at baseline as a predictor of response and survival (Table 2). All included studies used the Platelia™ Aspergillus antigen assay, although at different cut-offs. All studies included adult patients with proven and probable IA, unless stated otherwise in the table. We could not identify conflicting results between the articles: both statistically significant results and non-significant trends pointed in the same direction.

**Table 2:** Studies reporting statistics on a relation between baseline serum galactomannan and outcome.

**References**	**Population**	***N***	**Parameter at baseline**	**Measured Outcome**	***p*-value**
Imbert et al., 2016^a^	SOT, hematological, solid tumor, ICU	40	sGM < 2.0	50% of day 90 survivors vs. 25% of day 90 non-survivors had sGM < 2.0 at baseline	0.19
Vehreschild et al., 2017^c^	Majority hematological	40	Mean sGM	0.9 in week 12 survivors vs. 4.3 in week 12 non-survivors	0.047
Jung et al., 2017^a^	SOT, hematological, AIDS, diabetes	102	sGM < 0.5	28% of day 30 survivors vs. 24% of day 30 non-survivors had sGM < 0.5 at baseline	0.81
			sGM < 0.5	51% of day 90 survivors vs. 41% of day 90 non-survivors had sGM < 0.5 at baseline	0.29
Neofytos et al., 2015	SOT, hematological, solid tumor	47	sGM < 0.5	OR 4.5 for good response at week 6	0.05
			sGM < 0.5	OR 7.0 for week 12 survival	0.02
López-Medrano et al., 2016	Kidney transplant recipients	112	Mean sGM	0.5 in week 6 survivors vs. 1.1 in week 6 non-survivors	0.024
			Mean BAL GM	1.0 in week 6 survivors vs. 6.5 in week 6 non-survivors	0.014
Heylen et al., 2015	Kidney transplant recipients	41	sGM	HR 1.371 for week 12 mortality	0.002
			BAL GM	HR 1.742 for week 12 mortality	0.243
Han et al., 2015	Pediatric hematological	45	Median sGM	0.46 in week 12 survivors vs. 1.21 in week 12 non-survivors	0.015
Teering et al., 2014^a^^,^^d^	Mixed ICU	44	Mean sGM	Correlated with hospital survival (exact statistic not reported)	NS
Russo et al., 2014	Hematological, solid tumor, COPD (all non-neutropenic)	27	Mean BAL GM	1.9 in week 6 survivors vs. 3.6 in week 6 non-survivors	0.02
Kim et al., 2014^e^	Hematological	391	sGM < 0.5	HR 2.28 for good outcome	0.026
Hoyo et al., 2014	SOT	24	sGM < 0.5	56% of day 30 survivors vs. 18% of day 3 non-survivors had sGM < 0.5 at baseline	0.021
Mikulska et al., 2013^f^	Allogeneic stem cell transplant recipients	57	sGM 0.5–0.99	HR 2.76 for day 42 mortality	NS
			sGM ≥ 2.0	HR 6.98 for day 42 mortality	NS
			sGM 0.5–0.99	HR 1.37 for day 180 mortality	NS
			sGM ≥ 2.0	HR 3.35 for day 180 mortality	NS
Fisher et al., 2013	Allogeneic stem cell transplant recipients	100	sGM ≥ 0.5	Adjusted HR 3.01 for week 6 respiratory mortality	0.038
			sGM ≥ 2.0	Adjusted HR 6.56 for week 6 respiratory mortality	0.003
			sGM ≥ 1.0	Adjusted HR 2.54 for day 180 respiratory mortality	0.024
			sGM ≥ 2.0	Adjusted HR 4.01 for day 180 respiratory mortality	0.003
			sGM < 1.0	Adjusted HR 2.12 for day 180 survival	0.024
			sGM < 2.0	Adjusted HR 4.08 for day 180 survival	0.002
Hadrich et al., 2012	Hematological	58	sGM	HR 1.044 for mortality	NS
Bergeron et al., 2012	Hematological	57	sGM	HR 1.25 for day 60 mortality	< 0.05
Koo et al., 2010	Hematological, SOT, solid tumor	93	sGM	Adjusted HR 1.25 for week 6 mortality	0.039
Boutboul et al., 2002^b^^,^^g^	Hematological	58	Mean sGM	Correlated with clinical response (exact statistic not reported)	NS

SOT, Solid organ transplantation; sGM, Serum galactomannan; OR, Odds ratio; HR, Hazard ratio; NS, Not significant;

a Modification of the 2008 EORTC-MSG classification criteria. N;

b Author's own classification criteria;

c Only caspofungin treated patients;

d Also included possible cases;

e Only possible/probable cases, exclusion of patients with renal or hepatic failure. Outcome was a composite of 5 criteria;

f Only probable cases;

g *Outcome assessment after at least 7 days (not further specified)*.

Overall, there was a strong and consistent correlation between the level of sGM and both short-term and long-term survival, from day 42 up to day 180. Indeed, a well performed prospective randomized trial comparing anidulafungin in combination with voriconazole to voriconazole alone found baseline sGM to be only one of three independent predictors of week 6 survival in multivariate analysis (Marr et al., 2015). Stratifying patients by baseline sGM positivity (using a cutoff of 0.5) divided patients in two groups, with sGM positive patients having significantly higher mortality (Fisher et al., 2013; Hoyo et al., 2014; Kim et al., 2014; Neofytos et al., 2015; Jung et al., 2017). Three groups determined a different cutoff of sGM ≥ 2.0 based on the Youden index or analysis of the area under the curve (Fisher et al., 2013; Mikulska et al., 2013; Imbert et al., 2016). When stratified by this cutoff, two studies found a trend toward higher 42 and 90 day all-cause mortality (Mikulska et al., 2013; Imbert et al., 2016), with another study showing a statistically significant difference for both 6 week respiratory mortality, 180 day respiratory mortality, as well as 180 day all-cause mortality (Fisher et al., 2013).

This relation demonstrates the interplay between two factors that determine the progression of fungal disease. As shown before, sGM correlates with fungal burden. As such, a higher fungal burden (or higher baseline sGM) can be expected to result in worse outcomes. On the other hand, there is the link between neutrophils and GM, with neutrophils being necessary for clearing both sGM as well as the fungus itself. Indeed, higher sGM at diagnosis have been shown to correlate with lower neutrophil counts (Jung et al., 2017).

One study also reported a significant link between BAL GM and week 6 survival (López-Medrano et al., 2016). However, the relation between BAL GM and outcome should be interpreted with caution as others could not replicate this finding. Of note, BAL GM testing depends on the site of infection, the site of sampling (sampling error), the non-standardized collection of BAL fluid, as well as on the portion of BAL fluid tested (Racil et al., 2011).

## Impact of GM kinetics on outcome

We identified 21 studies that looked at GM kinetics as predictor of response and survival. Four descriptive studies were excluded due to the lack of a statistical analysis (Kwak et al., 2004; Maertens et al., 2005; Suankratay et al., 2006; Lai et al., 2007). The remainder has been summarized in Table 3. All included studies used the Platelia™ Aspergillus antigen assay. All studies included adult patients with proven and probable IA, unless stated otherwise in the table.

**Table 3:** Studies reporting statistics on a relation between galactomannan evolution after diagnosis and outcome.

**References**	**Population**	***N***	**Kinetic parameter**	**Measured outcome**	***p*-value**
Vehreschild et al., 2017^d^	Majority hematological	40	Mean sGM at day 7	0.3 in week 12 survivors vs. 1.1 in week 12 non-survivors	0.354
			Mean sGM at day 14	0.3 in week 12 survivors vs. 1.3 in week 12 non-survivors	0.559
			Mean of (day 14 sGM – day 7 sGM)	1.26 in week 12 survivors vs. 0.82 in non-survivors	0.617
Neofytos et al., 2015	SOT, hematological, solid tumor	47	Baseline sGM – Week 2 sGM	Mean difference 0.58 between week 6 responders vs. week 6 non-responders	0.03
			Baseline sGM – Week 6 sGM	Mean difference 0.65 between week 6 responders vs. week 6 non-responders	0.03
			Baseline sGM – Week 2 sGM	Mean difference 0.72 between week 12 responders vs. week 12 non-responders	0.02
			Baseline sGM – Week 6 sGM	Mean difference 0.98 between week 12 responders vs. week 12 non-responders	0.01
			sGM remaining < 0.5	OR 4.1 for week 6 response	0.07
			sGM remaining < 0.5	OR 4.5 for week 12 response	0.05
			sGM remaining < 0.5	OR 4.3 for week 6 survival	0.10
			sGM remaining < 0.5	OR 6.5 for week 12 survival	0.02
Han et al., 2015	Pediatric hematological	45	Week 1 median sGM	0.39 in week 12 survivors vs. 1.64 in week 12 non-survivors	0.015
			Week 2 median sGM	0.38 in week 12 survivors vs. 2.76 in week 12 non-survivors	0.004
			Week 1 sGM < 1.5	Predicts week 12 survival with sensitivity 61.5%, specificity 89.3%, NPV 83.3%, PPV 72.7%	
Teering et al., 2014^a^^,^^e^	Mixed ICU	44	Maximum sGM – baseline sGM	0.11 in in-hospital survivors vs. 0.48 in non-survivors	0.017
Chai et al., 2014	Majority hematological	147	Week 1 sGM – baseline sGM	Greater decline in week 12 responders in voriconazole treated patients (effect size not reported)	0.001
			Week 2 sGM – baseline sGM	Greater decline in week 12 responders in voriconazole treated patients (effect size not reported)	0.046
			Week 4 sGM – baseline sGM	Greater decline in week 12 responders in amphotericin B treated patients (effect size not reported)	0.072
Khanna et al., 2013^f^	Adults and children, no pathology specified	57	Increasing sGM	5.4% of day 30 survivors vs. 64.9% of day 30 non-survivors had increasing sGM	0.02
Nouér et al., 2012	Multiple myeloma	98	sGM < 0.5 within 7 days	Adjusted OR 2.9 for favorable week 6 response	0.048
			sGM < 0.5 within 7 days	45.5% of week 6 survivors vs. 22.6% of week 6 non-survivors had sGM < 0.5 within 7 days	0.03
			sGM < 0.5 within 7 days	Adjusted OR 2.9 for week 6 survival	0.048
Hadrich et al., 2012	Hematological	58	7 × (Week 1 sGM – baseline sGM)/days between tests	HR 0.709 for mortality	NS
Bergeron et al., 2012	Hematological	57	sGM area under the curve	No association found with day 60 survival	
			Rate of sGM decline	No association found with day 60 survival	
Park S. H. et al., 2011	Hematological	58	sGM remaining > 0.5 for more than 2 weeks	Kappa coefficient 0.663 for week 6 clinical failure	<0.05
			sGM remaining > 0.5 for more than 2 weeks	Kappa coefficient 0.819 for week 12 clinical failure	<0.05
Park S. Y. et al., 2011	Hematological, SOT	110	sGM remaining > 0.5 for more than 3 months	HR 7.14 day 90 mortality	<0.001
Nouér et al., 2011	Hematological	115	sGM remaining > 0.5 for more than 2 weeks	Kappa coefficient 0.819 for week 6 clinical failure	<0.001
Koo et al., 2010	Hematological, SOT, solid tumor	93	(Baseline sGM – week 1 sGM)/days between tests	Adjusted HR 0.78 for week 6 survival	0.02
Maertens et al., 2009^g^	Neutropenic hematological	70	sGM remaining > 0.5 for more than 2 weeks	Kappa coefficient 0.588 for week 6 clinical failure	<0.05
			sGM remaining > 0.5 for more than 2 weeks	Kappa coefficient 0.886 for week 12 clinical failure	<0.05
			sGM remaining > 0.5 for more than 2 weeks	Kappa coefficient 0.752 for week 6 EORTC-MSG response failure	<0.05
Woods et al., 2007^b^	Hematological	56	sGM remaining > 0.5 for more than 2 weeks	Kappa coefficient 0.861 for mortality	<0.0001
Boutboul et al., 2002^c^	Hematological	58	Increase of week 1 sGM < 1.0 over baseline	Predicts favorable week 6 response with sensitivity 44%, specificity 87%, PPV 94%	
			Increase of week 2 sGM < 1.0 over baseline	Predicts favorable week 6 response with sensitivity 55%, specificity 92%, PPV 92%	
Salonen et al., 2000^c^^,^^h^	Hematological, SOT	18	sGM remaining > 1.0	100% of non-survivors vs. 20% of survivors had sGM remaining > 1.0	0.002

SOT, Solid organ transplantation; sGM, Serum galactomannan; OR, Odds ratio; HR, Hazard ratio; NS, Not significant; PPV, Positive predictive value. N;

a Modification of the 2008 EORTC-MSG classification criteria;

b 2002 EORTC-MSG classification criteria;

c Author's own classification criteria;

d Only caspofungin treated patients;

e Also included possible cases;

f Interval between serial sGM assessments not specified;

g Only pulmonary invasive aspergillosis;

h *No interval specified between tests, or for outcome assessment*.

As with the baseline sGM, there appears to be a significant correlation between the evolution of sGM after baseline and outcome. Most studies stratified patients by outcome (treatment response or survival), and found significant differences in the mean sGM values at various timepoints (Woods et al., 2007; Maertens et al., 2009; Nouér et al., 2011, 2012; Park S. H. et al., 2011; Park S. Y. et al., 2011; Han et al., 2015; Neofytos et al., 2015; Vehreschild et al., 2017). The studies that took the initial sGM value into account and that evaluated the rate of decline, found this to be a good predictor of outcome as well (Boutboul et al., 2002; Koo et al., 2010; Khanna et al., 2013; Chai et al., 2014; Teering et al., 2014; Neofytos et al., 2015). For example, an increase of sGM values at week 2 of ≥1.0 over the baseline value, predicted therapy failure at week 6 with a sensitivity of 66%, a specificity of 87%, and a positive predictive value of 94% (Boutboul et al., 2002). The authors chose the cutoff of 1.0 as they determined this to be the smallest significant variance at higher optical indices. Furthermore, a persistently negative sGM was strongly associated with good outcomes (Neofytos et al., 2015). In another study, a composite of normalized serum 1,3-β-D-glucan (BDG, another biomarker of IA) and sGM (using z-scores) predicted clinical response at week 6 and week 12 (Neofytos et al., 2015). However, this appeared to be entirely due to the sGM kinetics as BDG alone failed to predict either, whereas sGM difference between baseline and week 2 predicted clinical response at week 6 and week 12. No study was able to identify differences in sGM before week 1.

Chai et al. found distinct kinetic profiles depending on the antifungal treatment, with voriconazole treatment showing earlier sGM clearance than amphotericin B treatment (Chai et al., 2014). However, this is in contrast with animal models where no difference in sGM kinetics could be seen between azole and polyene treatment (Petraitiene et al., 2001). Furthermore, another study in 93 patients found no differences in profiles between the antifungal drugs used (Koo et al., 2010).

## Impact of other biomarkers on outcome and survival

Besides GM, other quantitative biomarkers are being used for diagnosing IA such as BDG and *Aspergillus* PCR. These could therefore theoretically offer complementary information on prognosis and response to therapy as they have different sources of production and elimination. Indeed, a declining BDG at week 2 has been shown to correlate with survival at week 6 and week 12 (Neofytos et al., 2015). However, this decline was slower than the decline in sGM, and was less sensitive for predicting therapy response. The rate of decline seems to have an impact on survival however: a decline in BDG levels of 2.51 pg/mL/day had a sensitivity of 73.5% and specificity of 83.5% for predicting survival (Pini et al., 2016). Serum concentrations of bis(methylthio)gliotoxin (bmGT), a secondary metabolite of *Aspergillus* which has been proposed as a complementary biomarker, were shown to be significantly higher in patients who died at day 30 (2.36 ± 4.76 vs. 1.4 ± 7.58 mg/L, *p* < 0.01; Vidal-García et al., 2016).

In another study, a quantitative *Aspergillus* PCR showed good correlation between initial copy number and 90 day mortality, as well as between persistent PCR positivity after 2–3 weeks and 30 and 90 day mortality (Imbert et al., 2016). Similarly, a decline in circulating *Aspergillus* RNA between week 4 and week 6 correlated weakly with week 12 response (κ = 0.621, *p* = 0.026) but not with week 6 response (Zhao et al., 2016). A relation between sGM and circulating *Aspergillus* RNA could not be found. As such, these non-GM biomarkers appear to be especially useful in sGM negative patients, but are outperformed by sGM in sGM positive patients (which have a worse prognosis from the start), and only allow evaluation of antifungal efficacy during the later stages of treatment.

## What's next?

The data so far indicate a strong correlation between both baseline sGM and outcome, as well as between the kinetics of sGM and outcome. However, these correlations are based on average sGM values and offer little added value for the management of the individual patient, mainly due to the lack of specific thresholds. Therefore, several authors have proposed clinical decision rules based on their findings. However, validation of these proposed rules is lacking, both in the initial population from which these have been derived, as well as in external validation populations. As such, exact indicators of the accuracy, sensitivity, specificity and other parameters are not available, making these proposed decision rules not suitable yet for use in daily clinical practice. Furthermore, of the studies discussed above which used the Platelia™ Aspergillus ELISA, none addressed the issue of non-linearity of higher levels of sGM. Several studies have applied modifications of the EORTC-MSG consensus definitions, mostly including other host criteria such as AIDS, cirrhosis and chronic obstructive lung disease, and other clinical criteria, making comparison and interpretation of the results more difficult. In addition, many studies suffer from low to very low numbers of sGM samples per patient. This is sometimes circumvented by modeling the average kinetics of sGM in the population, and using this model to predict the expected value on a certain time point based on previous values. The resulting estimate is then used for further analysis. Both approaches are inherently subject to bias as the actual values at the time point of interest are unknown.

Currently, clinical trials evaluating antifungal drugs primarily use survival at week 6 or week 12 as the primary outcome, or the clinical response as defined in the EORTC-MSG criteria (Segal et al., 2008). Surrogate outcomes for earlier assessment of efficacy, which would potentially allow for shorter durations of clinical trials, have been proposed. One such endpoint defines success as repeatedly negative sGM (<0.5) for at least 2 weeks after the first negative sGM. This showed a good correlation with survival in 56 hematological patients (kappa correlation coefficient 0.861, *p* < 0.0001), which is in line with what would be expected from the kinetic data described above (Woods et al., 2007). This finding was confirmed by three independent studies in hematological patients, all of which found similar kappa correlation coefficients between this surrogate marker and clinical outcome and survival (Maertens et al., 2009; Nouér et al., 2011; Park S. H. et al., 2011). However, this definition does not allow evaluation of efficacy at a predetermined endpoint (e.g., after 1 or 2 weeks of treatment), which could be very useful in guiding decision making. In this setting, a robust and adequately validated early surrogate marker is not yet available.

Although the sensitivity of sGM for the diagnosis of IA is lower in non-neutropenic patients, solid organ transplant recipients, and patients on mold-active antifungal prophylaxis, the prognostic properties of sGM don't appear to be influenced by this. Several studies included non-neutropenic patients or solid organ transplant recipients (Koo et al., 2010; Park S. Y. et al., 2011; Russo et al., 2014; Teering et al., 2014; Neofytos et al., 2015; Imbert et al., 2016; Jung et al., 2017), or looked at these populations exclusively (Hoyo et al., 2014; Heylen et al., 2015; López-Medrano et al., 2016). The findings from these studies were in line with findings from studies in hematological patients. We could not identify any studies that looked at the difference in kinetics between patients on mold-active antifungal prophylaxis. However, several studies included this population in their overall analysis (percentage of study population on mold-active antifungal prophylaxis: range 4.3–85%, median 50%), and found results similar to those in populations not on prophylaxis (Park S. Y. et al., 2011; Hoyo et al., 2014; Kim et al., 2014; López-Medrano et al., 2016; Jung et al., 2017). We can thus conclude that patients with high initial sGM, and patients with an sGM that fails to decrease, are still at increased risk of poor outcome, independent of the underlying condition or prophylaxis. However, the exact kinetics could differ between these different populations, and have not been studied in detail.

## Conclusion

Baseline sGM and trends in sGM kinetics correlate with outcome (both response and survival) in IA. In addition, sGM appears to have early prognostic potential, especially in hematological patients. However, further studies are urgently needed to determine the precise clinically relevant breakpoints and their test characteristics, followed by validation in both hematological and non-hematological populations. Furthermore, several other biomarkers such as BDG, bmGT, and *Aspergillus* DNA or RNA, appear to offer additional and complementary information, although the amount of evidence for these biomarkers is as of yet sparse.

## Author contributions

TM was involved in data collection and drafting the article. TM, EG, KL, and JM were involved in critical revision of the article and final approval of the version to be published.

### Conflict of interest statement

TM has received lecture honoraria from Gilead and travel support from MSD and Gilead. JM has received research grants, travel support and lecture honoraria from Gilead, MSD, Basilea Pharmaceuticals, Astellas, and Pfizer and has participated in advisory boards for MSD, Gilead, Astellas, Basilea, Pfizer, F2G, Amplyx, Scynexis, and Cidara. KL has received research grants, travel support and lecture honoraria from Gilead, MSD and Pfizer. She participated in advisory boards for MSD and Gilead. The other author declares that the research was conducted in the absence of any commercial or financial relationships that could be construed as a potential conflict of interest.
